# A foot-care program to facilitate self-care by the elderly: a non-randomized intervention study

**DOI:** 10.1186/s13104-017-2898-9

**Published:** 2017-11-09

**Authors:** Shizuko Omote, Arisu Watanabe, Tomoko Hiramatsu, Emiko Saito, Masami Yokogawa, Rie Okamoto, Chiaki Sakakibara, Akie Ichimori, Kaoru Kyota, Keiko Tsukasaki

**Affiliations:** 1 Faculty of Health Science, Institute of Medical, Pharmaceutical and Health Sciences, Kanazawa University, 5-11-80 Kodatsuno, Kanazawa, Ishikawa 920-0942 Japan; 2 Division of Health Science, Graduate School of Medical Science, Kanazawa University, 5-11-80 Kodatsuno, Kanazawa, Ishikawa 920-0942 Japan; 3 School of Nursing, Kanazawa Medical University, 1-1 Daigaku, Uchinada, Ishikawa 920-0293 Japan; 4 Graduate School of Human Health Sciences, Tokyo Metropolitan University, 7-2-10 Higashiogu, Arakawa, Tokyo, 116-8551 Japan

**Keywords:** Program, Foot care, Self-care, Elderly, Support, Community

## Abstract

**Objective:**

We aimed to evaluate a foot-care awareness program designed to improve foot morphology, physical functioning, and fall prevention among the community-dwelling elderly. Eleven independent community-dwelling elderly women (aged 61–83 years) were provided with foot-care advice and shown effective foot-care techniques to perform regularly for 6 months, and compared with a control group of 10 elderly women who did not receive any intervention. Measurements of foot form, functional capacity, subjective foot movement, and physical function were taken at baseline and 6-month follow-up.

**Results:**

At follow-up, improvements were seen in the intervention group in foot morphology, subjective foot movement, foot pressure, and balance. In the intervention group, 90% of women had maintained or improved foot form and none of them had fallen during the post-intervention period, compared to the control group where 30% improved foot form (p = 0.0075) and four (40%) of them had fallen. Therefore, a foot-care program may have the potential to prevent falls and improve mobility among the elderly.

*Trial Registration* UMIN-CTR No. UMIN000029632. Date of Registration: October 19, 2017

**Electronic supplementary material:**

The online version of this article (10.1186/s13104-017-2898-9) contains supplementary material, which is available to authorized users.

## Introduction

The elderly are more likely to suffer from lower levels of self-care, increasing the chances of foot problems [1]. Keeping feet in good condition is essential for effective functioning, promoting leg strength, and reducing falls [2, 3]. Much research has examined attributes of the foot in relation to adverse health consequences. For example, foot posture is linked to lower extremity joint pain [4], whilst toe muscle strength is associated with balance and falls among the elderly [5]. Studies on foot care by medical professionals for the dependent elderly have found improvements in blood circulation, muscle fatigue, and walking ability [6, 7]. In a randomized controlled trial, Waxman and colleagues found elderly podiatry patients who received self-management foot-care instruction had lower foot disability scores at follow-up compared to those receiving usual care [8]. Many studies have focused on the elderly with medical conditions such as diabetes or rheumatoid arthritis [9–11]. Less is known about foot care carried out by the healthy elderly themselves. Evidence suggests that those who receive regular instructions for foot care at home continue the self-care with beneficial results [12]. As the healthy elderly have a reported poor awareness of foot care [13], there is a clear need for preventative self-care programs. To our knowledge, no intervention studies have examined the impact of foot-care instruction amongst healthy elderly individuals living independently.

We aimed to assess the effectiveness of a foot-care awareness program for the healthy elderly, and compare participants with a control group that did not receive foot-care instruction. We assessed whether the program improved foot morphology, physical functioning, and reduced falls, and examined whether it could be used independently without specialist instruction.

## Main text

### Methods

#### Participants

Twenty-seven participants were recruited from a Community Comprehensive Care Center that provides health support for the elderly in Kanazawa, Ishikawa prefecture, Japan. Individuals attending a dementia prevention workshop were invited to participate. Inclusion criteria were healthy individuals aged 60 and over who required no support for daily living, could carry out their own foot care, and lived in the jurisdiction of the care center. “Foot care” in this study refers to foot examination, cleaning, massage, and toe exercises carried out independently on a daily basis.

#### Ethical considerations

Participants were informed that participating or leaving the study would not result in any disadvantage and that personal information would be protected. We obtained written consent from all participants. We offered all members of the control group the full foot-care program at the end of the study. The Kanazawa University Medical Ethics Committee approved the study (approval# 478).

#### Intervention and control groups

The intervention group comprised of 17 women attending the dementia workshop in 2013. They attended lectures and practice sessions by nurses and a physiotherapist in the research team and were asked to perform foot care at home for 6 months whilst receiving support and periodic status check-ups. The control group were ten women of a similar age who had attended the same dementia workshop in 2011 and 2012, and were attending a further workshop in 2013. The study was conducted between December 2013 and June 2014.

#### Program content

##### The self-sufficiency course

A 90-min lecture/practice session was held weekly for two continuous weeks for those in the intervention group. The researchers created the program content with reference to foot anatomical physiology, foot-care publications, and other resources [14, 15]. Session one explained foot structure and function and practiced foot-care examination. Session two explained foot-care techniques and included a further practice session. Foot-care techniques were a combination of existing methods [15, 16] and resources from the Japanese Society for Foot-care covering: examination, washing, nail-clipping, toe exercise, massage, and appropriate footwear. A one-page instruction sheet on massage techniques and toe exercises was created to refer to. We encouraged participants to perform foot care at least weekly, as recommended by professionals [15, 16], and to complete a foot-care calendar to record when foot examination, massage, and toe exercises were carried out.

##### Periodic status check/support

A content-repetition lecture for ensuring foot-care awareness was held after 3 months. The intervention group received monthly phone calls for support, check foot-care status, and collect calendar entries.

##### Evaluation

Evaluation was carried out 6 months before and after the program implementation. Demographic information was collected from participants in the intervention and control groups on: gender, age, living circumstances, hospital attendance, and visibility levels. All participants were asked whether they could walk unaided for 15 min continuously and if any falls had occurred in the past 6 months. Daily life skills were assessed using the 13-item Tokyo Metropolitan Institute of Gerontology Index of Competence (TMIG-IC), to measure independence, intellectual activity, and social role. A point is scored for each item with higher scores indicating greater functional capacity.

Foot morphology and plantar pressure distribution were measured using the Foot View Clinic device (Nitta Corporation, Osaka, Japan). Nine subjective foot movements were examined: toe movement, toe spreading, balance when walking/standing, stumbling, foot lift when climbing stairs, tiredness when walking, foot ground contact sensation, coldness in feet, and foot cramps [6, 13]. These were rated on a four-point scale with a higher score representing worse foot movement.

Foot type was categorized as: standard (grounded foot); weakened (flat foot tendency); flat; hollow; and supinated (highly pressurized state on the heel and fifth toe) [17]. Changes in subjective foot movement and foot type at follow-up were evaluated as “improved,” “maintained,” “unchanged,” or “worsened.”

Physical function was assessed by: (1) walking ability –speed of walking 14 meters in a straight line; (2) muscle strength (toe-grip power)—seated participants gripped the researcher’s finger with their first and second toe without heel-lift and the grip was measured using a digital grip force meter (Takei Instrumentation Industry Co., Ltd.); (3) functional reach—subjects raised their arms with both feet spread and leant forward without heel-lift to press a bar attached to a ruler (Aussie Co., Ltd). The distance of the bar was then measured, taking into account the participant’s age.

Members in the intervention group assessed the program utilizing a four-point scale to rank the opening and follow-up lectures, and the monthly support checks.

##### Statistical analysis

Foot-care implementation rate was calculated by dividing the number of days checked on the calendar by the number of days during the period. McNemar’s test compared data before and after the program implementation. Chi square tests compared the measurements between the intervention and control groups. Wilcoxon signed-rank tests compared measurements at baseline and follow-up for the intervention group and functional reach measurements for both intervention and control groups. T-tests compared pressure point measurements in the intervention group at baseline and follow-up. Significance was set at 5%. We used SPSS version 22 (Chicago, IL) for the analysis.

### Results

#### Baseline characteristics

Eleven of the 17 participants were included in the intervention group; six discontinued because of personal/family sickness or time constraints. All participants were women, with an average age of 71.8 years (SD ± 7.1) in the intervention group and 74.3 (SD ± 4.7) in the controls. Approximately 80% of participants in both groups exercised regularly. The average TMIG-IC score was 11.9 ± 1.2 and 12.1 ± 1.4 in the intervention and control groups respectively, indicating good functional capacity. All participants in the intervention group and 90% of the control group could walk unaided for 15 min continuously. One participant in each group had fallen in the 6 months before implementation. There were no further falls in the intervention group in the subsequent 6 months whilst four (40%) of the control group had fallen.

#### Foot-care implementation

Nine participants carried out foot care at least weekly. The average ratio of implementation was 65.6% (5 days/week) for foot observation, 61.8% (4–5 days/week) for foot massage, and 70.6% (5–6 days/week) for toe exercises.

#### Foot morphology/plantar pressure distribution

In the intervention group, one participant’s foot morphology was flat-footed at baseline but this had resolved 6 months later. Two participants with plantar pressure biased to the outer foot showed front/rear pressure distribution at follow-up, with one developing a visible medial longitudinal arch. Two participants with lower pressure on the first toe versus other toes improved 6 months later (Table 1).

**Table 1:** Plantar pressure balance in intervention and control groups

Pressure point	Intervention group (n = 11)	Baseline^a^		6 month follow-up^b^		a:b^2^ *p* value
	Control group (n = 10)	Mean ± SD	*p* value^1^	Mean ± SD	*p* value^1^	
Right	Intervention	48.8 ± 4.8	0.63	51.5 ± 7.1	0.22	0.12 0.94
	Control	47.7 ± 5.6		47.8 ± 6.4		
Front right	Intervention	39.5 ± 9.2	0.27	43.7 ± 10.5	0.83	0.13 0.69
	Control	43.5 ± 6.6		44.7 ± 9.4		
Front left	Intervention	42.7 ± 11.9	0.95	47.6 ± 11.0	0.49	*0.05*
	Control	42.4 ± 11.2		43.7 ± 14.2		0.73
Left	Intervention	51.2 ± 4.8	0.63	48.5 ± 7.1	0.22	0.12
	Control	52.3 ± 5.6		52.2 ± 6.4		0.94
Right rear	Intervention	60.5 ± 9.2	0.25	56.3 ± 10.5	0.83	0.13
	Control	56.7 ± 6.8		55.3 ± 9.4		0.64
Left rear	Intervention	52.3 ± 11.9	0.95	52.5 ± 11.0	0.49	*0.05*
	Control	57.6 ± 11.3		56.3 ± 14.2		0.73

^1^ t-test

^2^ paired t-test

^a^Baseline

^b^6 month follow-up

#### Subjective foot movement

Ten participants improved or maintained toe movement and toe spreading, nine improved or maintained foot ground contact sensation, and eight maintained or improved stability of walking/standing balance and feet lift when climbing stairs (Additional file 1). However, these changes were not significantly different from controls.

#### Foot-care practice

At follow-up, all foot-care practices had increased in the intervention group compared with baseline, except applying foot cream and reporting an interest in foot care, which remained unchanged (Additional file 2). However, these differences were not statistically significant, nor was there a significant difference between groups in carrying out foot care.

#### Comparison of foot form

**Fig. 1:**
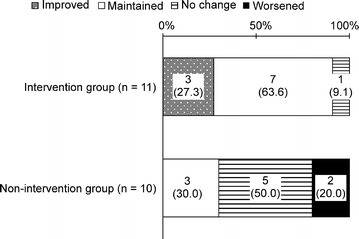
Change in foot form in the intervention and control groups

Ten (90.9%) people’s foot form in the intervention group had either maintained or improved at follow-up (Fig. 1). This improvement was significantly greater than the controls, of whom three (30.0%) had maintained/improved foot form (p = 0.0075).

#### Physical function

There were no statistically significant differences at baseline and follow-up in the intervention group in improved grip strength, right toe pressure, functional reach, or walking speed (Table 2).

**Table 2:** Changes in physical function in intervention and control groups

Function	Intervention group (n = 11)	Baseline time mean ± standard deviation	Baseline group comparison *p* value^1^	After 6 months mean ± standard deviation	Comparison between groups after 6 months *p* value^1^	Comparison between baseline and after 6 months based on group *p* value
	Control group (n = 10)					
Left grip strength (kg)	Intervention group	21.3 ± 4.1	0.28^1^	21.5 ± 3.8	0.52^1^	0.56^2^
	Control group	19.3 ± 3.8		20.5 ± 3.0		0.072
Right grip strength (kg)	Intervention group	21.6 ± 3.5	0.33^1^	21.4 ± 4.4	0.70^1^	0.80^2^
	Control group	20.3 ± 2.7		20.8 ± 3.4		0.42^2^
Grip between left foot toes (kg)	Intervention group	2.6 ± 0.7	0.73^1^	2.2 ± 0.8	0.61^1^	0.03*^2^
	Control group	2.7 ± 0.8		2.3 ± 0.6		0.03*^2^
Grip between right foot toes (kg)	Intervention group	2.8 ± 0.5	0.86^1^	2.7 ± 0.7	0.28^1^	0.75^2^
	Control group	2.7 ± 0.8		2.4 ± 0.5		0.08^2^
FRT age ratio (%)	Intervention group	104.0 ± 11.2	0.67^3^	104.5 ± 9.8	0.90^1^	0.18^4^
	Control group	101.1 ± 20.1		105.5 ± 22.1		0.34^2^
14-m 10 walk (s)	Intervention group	6.9 ± 0.9	0.42^1^	7.2 ± 1.1	0.41^1^	0.23^2^
	Control group	7.2 ± 0.8		7.6 ± 1.1		0.14^2^

^1^t-test

^2^Paired t-test

^3^Wilcoxon rank sum test

^4^Wilcoxon signed-rank test

* Significant difference between baseline and follow-up

#### Participants’ evaluation

All participants in the intervention group reported that the lecture and monthly phone calls were beneficial. Two participants who did not make foot-care calendar entries said it was “too troublesome” but responded that they had carried out foot care at least once a week.

## Discussion

We found changes in foot morphology occurred in all participants 6 months after foot care implementation. No falls had occurred in the intervention group, while 40% of controls had fallen. In particular, increased plantar pressure was observed on the first toe compared to other toes, which is important for effective “kick-out” force whilst walking. These foot changes may be due to improved strength from toe exercising, supporting earlier work that strengthening toe flexor muscles can significantly affect balance ability and reduce falls [5]. Other fall prevention programs have also shown positive results through lower extremity exercises [18]. We found changes in some individuals’ center of gravity and arch of the foot, which are important for balance and stability [19]. Regarding subjective foot movement, 90% of participants showed improved/maintained toe movement and toe spreading, while 70% showed improved/maintained foot ground contact sensation and stability of walking/standing balance. An association between toe strength and balance has been reported previously. Nagai et al. [20] found toe and ankle training in those living in nursing homes significantly improved balance ability and reduced the fear of falling.

All participants carried out foot care at least weekly and reported benefits from the mid-term lecture and phone calls. Previous research has shown telephone intervention by nurses is effective in heightening treatment adherence [21]. We believe the supportive telephone calls resulted in participants averaging more than 4 days a week of foot-care. Further, given the greater continuance and higher rate of implementation compared with previous studies [12], our evaluation suggests that this program increases self-care among the elderly and helps maintain and improve independent walking ability.

To conclude, we have shown that foot-care instruction can benefit the independent elderly, particularly in maintaining foot form and improving foot pressure and balance, which may have important implications in fall prevention.

## Limitations

The principal limitation of this study is its observational nature and the non-randomization of participants. Therefore, while we showed an association between the program and health improvements, we cannot determine if this was fully related to the intervention. The study included a small number of participants. This is similar to small-scale studies on lower extremities, including fall prevention in the elderly [18, 22–24]. We did not conduct blind assessment of treatment effects, which may have introduced bias. Our sample included highly able and active elderly people, which may explain the lack of significant improvements in foot morphology and subjective foot movement. We are also unable to generalize our findings to elderly men. Future work should consider the effects of the program at longer follow-up periods, and without support and monthly encouragement.

## Additional files

**Additional file 1.** Subjective foot movement in intervention and control groups.

**Additional file 2.** Foot-care practice in intervention and control groups.

## Abbreviation

TMIG-IC: Tokyo Metropolitan Institute of Gerontology Index of Competence
