# Supplementary material for: A foot-care program to facilitate self-care by the elderly: a non-randomized intervention study
Source: BMC Res Notes. 2017 Nov 9;10:586. doi: 10.1186/s13104-017-2898-9 (PMC5679143; doi:10.1186/s13104-017-2898-9)
Supplement: Supplementary file 1 — Additional file 1. Subjective foot movement in intervention and control groups. [file 13104_2017_2898_MOESM1_ESM.docx]

**Additional file 1.** Subjective foot movement in intervention and control groups.

| Movement | Intervention group (n=11) | Baseline^~~a~~^ | |  | Six-month  follow-up^~~b~~^ | | Comparison between baseline and at 6 months |
| --- | --- | --- | --- | --- | --- | --- | --- |
|  | Control group (n=10) | N (%) | *P*-value^1^ |  | N (%) | *P-*value^1^ | *P*-value² |
| Toe movement | Intervention  Control | 9 (81.8)  10 (100) | - |  | 10 (90.9)  9 (90.0) | 1.00 | 1.00  - |
| Toe spreading | Intervention  Control | 9 (81.8)  10 (100) | - |  | 10 (90.9)  9 (90.0) | 1.00 | 1.00  - |
| Stability of walking/ standing balance | Intervention  Control | 9 (81.8)  9 (90) | 1.00 |  | 8 (72.7)  10 (100.0) | - | 1.00  - |
| Stumbling while walking | Intervention  Control | 5 (45.5)  5 (50.0) | 1.00 |  | 4 (36.4)  4 (40.0) | 1.00 | 1.00  0.50 |
| Feet lift when climbing stairs | Intervention  Control | 10 (90.9)  9 (90.0) | 1.00 |  | 10 (90.9)  9 (90.0) | 1.00 | 1.00  1.00 |
| Tiredness while walking | Intervention  Control | 6 (54.5)  6 (60.0) | 1.00 |  | 6 (54.5)  6(60.0) | 1.00 | 1.00  0.73 |
| Foot ground contact sensation | Intervention  Control | 10 (90.9)  10 (100.0) | - |  | 10 (90.9)  10 (100.0) | - | 1.00  - |
| Sensation of coldness in feet | Intervention  Control | 6 (54.5)  7 (70.0) | 0.66 |  | 6 (54.5)  8 (80.0) | 0.36 | 1.00  - |
| Foot cramps | Intervention  Control | 6(54.5)  7 (70.0) | 0.66 |  | 7 (63.6)  8 (80.0) | 0.64 | 1.00  1.00 |

^1^χ² test　^2^McNemar's test
