# Supplementary material for: A foot-care program to facilitate self-care by the elderly: a non-randomized intervention study
Source: BMC Res Notes. 2017 Nov 9;10:586. doi: 10.1186/s13104-017-2898-9 (PMC5679143; doi:10.1186/s13104-017-2898-9)
Supplement: Supplementary file 2 — Additional file 2. Foot-care practice in intervention and control groups. [file 13104_2017_2898_MOESM2_ESM.docx]

**Additional file 2.** Foot-care practice in intervention and control groups.

| Practice | Intervention group (n=11) | Baseline^a^ | |  | Six-month  follow-up^b^ | | Comparison between baseline and at 6 months |
| --- | --- | --- | --- | --- | --- | --- | --- |
|  | Control group (n=10) | N (%) | *P*-value^1^ |  | N (%) | *P*-value^1^ | *P*-value² |
| Washing between the toes | Intervention  Control | 9 (81.8)  10 (100) | - |  | 10 (90.9)  10 (100.0) | - | 1.00  - |
| Examining feet | Intervention  Control | 6 (54.5)  9 (90.0) | 0.15 |  | 9 (81.8)  9 (90.0) | 1.00 | 0.25  1.00 |
| Clipping the toenail(s) in a square | Intervention  Control | 2 (18.2)  0 (0.0) | - |  | 3 (27.3)  1 (10.0) | 0.59 | 1.00  - |
| Taking care of the cuticles | Intervention  Control | 5 (45.5)  6 (60.0) | 0.67 |  | 7 (63.6)  6 (60.0) | 1.00 | 0.50  1.00 |
| Applying cream to the feet | Intervention  Control | 5 (45.5)  7 (70.0) | 0.38 |  | 5 (45.5)  8 (80.0) | 0.18 | 1.00  1.00 |
| Massaging the feet³ | Intervention  Control | -  - | - |  | 7 (63.6)  7 (70.0) | 1.00 | -  - |
| Performing toe exercise | Intervention  Control | 5 (45.5)  9 (90.0) | 0.06 |  | 9 (81.8)  7 (70.0) | 0.64 | 0.13  0.50 |
| Wearing shoes that fit | Intervention  Control | 9 (81.8)  10 (100.0) | - |  | 11 (100.0)  9 (90.0) | - | -  - |
| Being interested in foot care | Intervention  Control | 9 (81.8)  9 (90.0) | 1.00 |  | 9 (81.8)  8 (80.0) | 1.00 | 1.00  1.00 |

^1^χ² test　^2^McNemar's test ^3^At 6 months
